# Report of the Diseases of Edinburgh for March, 1810

**Published:** 1810-05

**Authors:** John Roberton


### *Report of the Diseases of Edinburgh for March,* 1810.
### By John Roberton, M. D.

The weather, on the commencement of the month, was for some days cloudy, and we had several showers of rain. In addition to this state of the weather, we had, till the middle of the month, occasional falls of snow, with some intervening clear days. The weather then became pleasant, and continued so till within a few days of the termination of the month, when it again became cloudy, and so continued till the end.

The Barometer continued rather low during the whole month.

The Thermometer stood about 40, or between that and 35 during most part of the month. Toward the end, however, it was sometimes as high as 55.

The diseases which have been most common were chincough, some cases of measles, inflammatory complaints in general, severe cases of diarrhœa, often attended with an affection of the stomach, chronic catarrh, and fevers of various degrees of violence; some of them of a bad kind, running their course in a few days, while, in many instances, some were protracted a very long time.

Chin-cough has greatly increased since last Report, both in extent and in severity. There are parts of the City so completely infested by it, that almost every infant has been affected by that distressing malady. In the present, as in most instances of contagious disease, I have been at some trouble in endeavouring to trace the place where it first originated. The result of my investigations on this subject, I have detailed in my lately published work on Medical Police, &c. &c.; therefore I shall not now dwell upon it. I have in these investigations always found that those diseases originated in low ill-aired places, and, in the present instance, have traced it to Canal Street, situate on the banks of the remaining marshes of the North Loch. This nuisance, though much diminished of late years, still continues to vitiate the surrounding atmosphere, and with the assistance of the putridity generated in the shambles, almost immediately in the same neighbourhood, probably gave rise to the disease. This method of attempting to account for the generation and propagation of these always distressing, and too often fatal diseases, carries with it proofs of a seemingly incontrovertible kind. It teaches us that cleanliness ought not only to be considered as an ornament to our persons, but highly beneficial; not only in the prolongation of our lives, but also of those who, although at some distance from such places, may have their health injured by their existence.

The measles have not increased either in extent or severity since last month, neither do they seem to have abated, as in various families the disease is to be found.

Although inflammatory complaints have neither been so general nor so severe during the winter, as in almost every previous year, yet there has constantly existed a sort of inflammatory diathesis, which from time to time has appeared in various forms, and under various states of severity. Indeed, although none of these inflammatory affections have individually been very widely diffused; yet there is, perhaps, scarcely one variety of them in the whole of Cullen's arrangement, but has been met with. Thus it would appear, that what we have gained by their severity being less conspicuous and less fatal, we have lost by the number of such affections being increased, and consequently, by the general state of health being more widely disordered.

There have appeared some very severe cases of diarrhœa, of a degree of obstinacy much beyond what we usually meet with in practice. Although many cases of this disease seemed to owe their existence principally to a disordered state of the intestinal canal, independently of any affection of the stomach, yet in a great variety of instances, it was accompanied by an evident affection of that organ. In the first, purgative medicines alone, with, in a few instances, the use of the warm bath, entirely removed them. But in the other, which was often accompanied by severe head-ach, pain of stomach, with frequent and severe sickness and violent retching, the practice necessary for their removal required much greater discrimination. In many of them purgatives were, on their administration, instantly vomited, and this often gave increase to the retching, which before was much less violent. On such occasions too the pain of head and stomach exceeded any thing that can be described. In these cases, although pain in the head head was certainly only a consequence of the affection of the stomach, I have found it necessary, even for momentary relief, to apply leeches to the temples, and even in some cases to open the jugular vein or temporal artery. After this, or at the same instant, to apply a large blister over or even beyond the whole surface occupied by the stomach. I have found this practice, even previous to the use of any internal medicine, permit us to administer such medicines; and such practice, ultimately effected a perfect cure. In others, however, the stomach has continued so irritable, that unless purgatives were, at every dose, accompanied by a small proportion of extract of hyosciamus, they were instantly vomited. In addition to all these plans of practice I have, in some cases, found it absolutely necessary to order an anodyne glyster. By patient perseverance in one or more of these methods of cure, according to the severity of the case, they have all yielded, and the patient's health has been ultimately restored.

I have frequently had occasion to take notice of a sort of chronic catarrh, very common, I believe, throughout most of the British Empire. It is usually the sequela of ill-treated or neglected catarrh, when it appears in its acute state. And not uncommonly, without being preceded by acute catarrh, it affects old and debilitated persons, and proves, especially in the morning, exceedingly troublesome to them. This, from either of these causes, is not an easily cured affection, but it may in almost every instance be greatly relieved. In my former reports, as well as in my treatise on Medical Police, there will be found a full account of this disease, and of the means which, in many instances, I have found best calculated for the alleviation of its symptoms.

Fevers still of the typhoid kind, prevail rather extensively. Some of them are much worse than others, and run their course much more rapidly. In various instances they have, independently of every attention, terminated fatally in a few days, while in others, much more numerous than these mentioned, their course seems to be protracted often for several weeks, nor do such cases seem to terminate so fatally as the former. To prevent disease ought always to be our first object, and to cure it our next. More attention being paid to the first of these than is usually done, would soon render the necessity of attention to the second much less. In no disease can this observation be more justly applied than to the various kinds of febrile diseases, and in none has it been less attended to.

*Princes Street.*

